# Aflibercept and Brolucizumab in Diabetic Macular Edema: A Focused Review of Efficacy, Safety, and Clinical Considerations

**DOI:** 10.3390/biomedicines14030501

**Published:** 2026-02-25

**Authors:** Ana Maria Dascalu, Catalin Cicerone Grigorescu, Ece Ergin, Cristina Alexandrescu, Dan Dumitrescu, Marina Ionela Nedea, Bogdan Mihai Cristea, Dragos Serban, Laura Carina Tribus, Tudor Mihai Badescu

**Affiliations:** 1 Doctoral School, “Carol Davila” University of Medicine and Pharmacy Bucharest, 020021 Bucharest, Romania; ana.dascalu@umfcd.ro (A.M.D.); dragos.serban@umfcd.ro (D.S.); 2 Ophthalmology Department, Emergency University Hospital Bucharest, 050098 Bucharest, Romania; 3 Faculty of Medicine, “Carol Davila” University of Medicine and Pharmacy Bucharest, 020021 Bucharest, Romania; bogdan.cristea@umfcd.ro; 4 Facultyof Medicine, “Vasile Goldis” Western University of Arad, 310130 Arad, Romania; grigorescu.cicerone@uvvg.ro; 5 Faculty of Pharmacy, “Carol Davila” University of Medicine and Pharmacy Bucharest, 020021 Bucharest, Romania; 6 Faculty of Dental Medicine, “Carol Davila” University of Medicine and Pharmacy Bucharest, 020021 Bucharest, Romania; 7 Faculty of Medicine, “Lucian Blaga” University of Sibiu, 550169 Sibiu, Romania; tudor.badescu@ulbsibiu.ro

**Keywords:** diabetic retinopathy, diabetic macular edema, anti-VEGF therapy, aflibercept, brolucizumab, intravitreal injections

## Abstract

**Background/Objectives:** Diabetic macular edema (DME) are major causes of visual impairment worldwide, with vascular endothelial growth factor (VEGF) playing a central role in disease pathogenesis. Intravitreal anti-VEGF therapy is the current standard of care for center-involving DME. **Methods:** This narrative review summarizes and compares the pharmacological properties, clinical efficacy, safety, and real-world performance of aflibercept and brolucizumab in the treatment of DME, based on randomized controlled trials and observational studies published between 2014 and 2025. **Results:** Both agents demonstrate significant improvements in visual acuity and retinal anatomy. Brolucizumab has shown non-inferior visual outcomes and, in several studies, greater reductions in central retinal thickness with the potential for extended dosing intervals, suggesting reduced treatment burden. However, post-marketing data have identified an increased risk of intraocular inflammation, including retinal vasculitis and retinal vascular occlusion, associated with brolucizumab, which has influenced its clinical use. **Conclusions:** Both aflibercept and brolucizumab are effective anti-VEGF therapies for diabetic macular edema, but they differ in durability and safety considerations. Aflibercept remains a reliable first-line option for many patients, while brolucizumab may be considered in carefully selected cases where treatment burden is a priority. Individualized treatment selection based on clinical characteristics, patient preferences, and safety considerations is essential for optimizing long-term outcomes.

## 1. Introduction

Diabetic retinopathy (DR) remains one of the leading causes of vision impairment and blindness among working-age adults worldwide [1,2]. As the global prevalence of diabetes continues to rise, the burden of DR and its vision-threatening complications, particularly diabetic macular edema (DME), is expected to increase substantially, posing significant challenges to patients, healthcare systems, and society [1,2,3].

The pathogenesis of DR is complex and multifactorial, involving chronic hyperglycemia-induced microvascular damage, inflammation, and progressive retinal ischemia [2,3,4]. Among the molecular mediators implicated in disease progression, vascular endothelial growth factor (VEGF) plays a central role by promoting increased vascular permeability and pathological angiogenesis [3,4,5]. Elevated intraocular VEGF levels have been consistently associated with the development of DME and proliferative diabetic retinopathy, providing a strong biological rationale for targeted VEGF inhibition [5,6,7].

The intravitreal anti-VEGF therapy has fundamentally transformed the management of diabetic retinal disease. Compared with conventional treatments such as focal or grid laser photocoagulation, anti-VEGF agents have demonstrated superior visual and anatomical outcomes and have become the standard of care for center-involving DME and for selected cases of diabetic retinopathy [8,9,10,11,12]. Nevertheless, the chronic nature of DR necessitates repeated intravitreal injections and long-term monitoring, emphasizing the importance of treatment durability, safety, and real-world feasibility [10,11,12].

Multiple anti-VEGF agents are currently available for the treatment of diabetic macular edema, including Aflibercept, Bevacizumab, Brolucizumab, Conbercept, Ranibizumab, and Pegaptanib, and more recently Faricimab [13,14]. While broad comparisons across all agents may provide a general overview of treatment efficacy, they often limit clinically practical interpretation. A more focused comparison between therapies with clearly distinct pharmacologic and clinical characteristics may therefore be more informative for decision-making in daily practice.

In this context, aflibercept and brolucizumab represent two agents with contrasting therapeutic profiles. Aflibercept is widely used as a reference anti-VEGF therapy supported by extensive long-term evidence [6,7,8,9,10], whereas brolucizumab was introduced with the aim of improving treatment durability but has raised safety considerations requiring careful patient selection [14,15,16]. In routine clinical practice in our region, these agents are frequently selected as first-line or switch options, making their comparison particularly relevant when balancing treatment burden against safety profile.

Given these differences, a comparative evaluation of aflibercept and brolucizumab is clinically relevant for optimizing individualized treatment strategies in patients with DME. This review aims to synthesize current evidence regarding the pharmacology, efficacy, safety, and real-world performance of these agents, with a focus on informing contemporary clinical decision-making.

## 2. Literature Search Strategy

A comprehensive literature review was conducted to evaluate and compare the efficacy and safety of aflibercept and brolucizumab in the treatment of DME. Searches were performed in PubMed, Web of Science, and Scopus databases for articles published between January 2014 and December 2025. Search terms included combinations of “diabetic macular edema,” AND “aflibercept” AND/OR “brolucizumab”. The paper aims to provide a narrative synthesis integrating evidence from randomized control trials (RCTs), extension studies, and real-world data to offer a clinically contextualized perspective. Relevant papers written in English, involving adult patients with DME that underwent intravitreal therapy with either aflibercept or brolucizumab were critically analyzed.

Case reports, conference abstracts without full text, non-English publications, and studies unrelated to diabetic macular edema were excluded. Reference lists of key review articles were also screened to identify additional relevant publications.

The research was conducted by 2 independent ophthalmologists, with special interest in diabetic retinopathy. Any disagreement was solved by discussion.

## 3. Pathophysiology of Diabetic Retinopathy and Rationale for Anti-VEGF Therapy

Diabetic retinopathy is a progressive microvascular complication of diabetes mellitus, driven by chronic hyperglycemia and resulting in structural and functional alterations of the retinal vasculature [1,2,3]. Prolonged metabolic dysregulation leads to endothelial dysfunction, loss of pericytes, basement membrane thickening, and disruption of the blood–retinal barrier, collectively contributing to increased vascular permeability, capillary nonperfusion and retinal ischemia [2,3,4,5].

Diabetic macular edema represents a principal cause of vision loss in diabetic retinopathy and arises primarily from breakdown of the blood–retinal barrier. Increased vascular permeability allows fluid and plasma constituents to leak into the retinal tissue, leading to retinal thickening and distortion of normal retinal architecture [3,5]. In parallel, progressive retinal ischemia promotes the release of angiogenic and inflammatory mediators that further exacerbate vascular instability and disease progression [5,6].

Among these mediators, vascular endothelial growth factor (VEGF) plays a central role in the pathogenesis of diabetic retinal disease. VEGF expression is upregulated in response to hypoxia and inflammatory signaling and contributes to both increased vascular permeability and pathological neovascularization [3,4,5,6]. Elevated intraocular VEGF levels have been consistently demonstrated in patients with diabetic macular edema and proliferative diabetic retinopathy and have been shown to correlate with disease severity [5,6].

In addition to VEGF-driven pathways, inflammation plays a significant role in the development and progression of diabetic retinopathy. Pro-inflammatory cytokines, chemokines, and adhesion molecules promote leukostasis, endothelial injury, and capillary occlusion, further impairing retinal perfusion [3,5,6]. The interplay between ischemia, inflammation, and VEGF signaling underscores the multifactorial nature of diabetic retinopathy and highlights the need for targeted therapeutic intervention.

The identification of VEGF as a key pathogenic factor provided a strong biological rationale for the development of intravitreal anti-VEGF therapies. By inhibiting VEGF signaling, anti-VEGF agents reduce vascular permeability, suppress neovascularization, and promote stabilization of the retinal vasculature, leading to improvements in retinal anatomy and visual function [8,9,10,11,12]. These therapeutic effects have been demonstrated consistently across randomized clinical trials and have established VEGF inhibition as a cornerstone of modern diabetic retinopathy management.

Despite the effectiveness of VEGF-targeted therapy, diabetic retinopathy remains a chronic and progressive disease, necessitating sustained treatment and long-term monitoring. A clear understanding of the underlying pathophysiology is therefore essential for interpreting differences in efficacy, durability, and safety among available anti-VEGF agents and for optimizing individualized treatment strategies.

### 3.1. Anti-VEGF Agents in Diabetic Retinopathy: An Overview

The introduction of intravitreal anti-VEGF therapy has fundamentally altered the management of diabetic retinopathy and diabetic macular edema. Prior to the anti-VEGF era, treatment strategies relied largely on focal or grid laser photocoagulation and, in advanced cases, vitrectomy. While these approaches reduced the risk of severe vision loss, they were limited in their ability to improve visual acuity and were associated with irreversible retinal damage [8,9,10].

Anti-VEGF agents directly target a central pathogenic pathway in diabetic retinal disease by inhibiting VEGF-mediated vascular permeability and angiogenesis. Multiple randomized clinical trials have demonstrated that anti-VEGF therapy results in superior visual acuity gains and greater anatomical improvement compared with laser therapy in patients with center-involving diabetic macular edema, establishing intravitreal anti-VEGF injections as the current standard of care [8,9,10,11,12].

Several anti-VEGF agents are approved for use in diabetic retinal disease, differing in molecular structure, binding properties, pharmacokinetics, and dosing regimens. Despite these differences, all approved agents aim to suppress intraocular VEGF activity, leading to reduced macular edema, improved visual acuity, and stabilization or regression of diabetic retinopathy severity in a subset of treated eyes [10,11,12]. Clinical studies have further suggested that sustained VEGF inhibition may modify the natural course of diabetic retinopathy by reducing disease progression [11,12].

The widespread adoption of anti-VEGF therapy has also highlighted challenges related to long-term disease management. Diabetic retinopathy is a chronic condition requiring repeated intravitreal injections and ongoing monitoring, which can impose a significant burden on patients, caregivers, and healthcare systems. In real-world practice, treatment adherence and injection frequency often fall short of clinical trial protocols, potentially compromising long-term outcomes [12].

Within this therapeutic landscape, aflibercept and brolucizumab represent two anti-VEGF agents with distinct clinical profiles. Aflibercept is supported by extensive evidence from randomized trials and real-world studies demonstrating consistent efficacy and long-term safety [8,9,10,11,12]. Brolucizumab was developed with the aim of achieving sustained VEGF suppression and extended dosing intervals and has demonstrated comparable efficacy in clinical trials, though safety considerations have influenced its clinical utilization [15,16,17]. This broader context provides the foundation for the detailed comparative evaluation of these agents presented in the following sections.

### 3.2. Pharmacological and Molecular Differences Between Aflibercept and Brolucizumab

Understanding the pharmacological and molecular characteristics of anti–vascular endothelial growth factor (anti-VEGF) agents is essential for interpreting differences in clinical efficacy, durability, and safety in the treatment of diabetic retinal disease. Although both aflibercept and brolucizumab target VEGF-A, they differ substantially in molecular structure, size, and binding properties, which may influence their intravitreal behavior and clinical performance.

Aflibercept is a recombinant fusion protein composed of extracellular domains of human VEGF receptor-1 and VEGF receptor-2 fused to the Fc portion of human IgG1. This molecular configuration enables aflibercept to bind VEGF-A, VEGF-B, and placental growth factor, providing broad inhibition of VEGF-mediated signaling pathways [18,19]. With a molecular weight of approximately 115 kDa, aflibercept exhibits well-characterized intravitreal pharmacokinetics that have been associated with consistent clinical efficacy across multiple retinal vascular diseases [19,20].

In contrast, brolucizumab is a humanized single-chain antibody fragment with a substantially smaller molecular weight of approximately 26 kDa. Its compact structure allows delivery of a 11 to 13 times higher molar concentration compared to aflibercept [18,19]. Brolucizumab demonstrates high binding affinity for VEGF-A and may achieve enhanced retinal tissue penetration, contributing to sustained VEGF suppression [20].

These molecular distinctions are believed to underpin differences in treatment durability observed in clinical studies. The higher molar dose and smaller molecular size of brolucizumab may facilitate longer dosing intervals in selected patients, whereas aflibercept has demonstrated durable efficacy with established dosing regimens across a broad patient population [20,21]. Such pharmacological properties are particularly relevant in the context of chronic diseases, such as diabetic retinopathy, where treatment burden is a key consideration.

However, molecular design may also influence immunogenicity. The unique structure of brolucizumab has been proposed as a potential factor contributing to the inflammatory events reported in post-marketing surveillance, whereas aflibercept has demonstrated a comparatively stable and well-characterized safety profile over long-term use [19,20,21]. These pharmacological differences provide an important mechanistic framework for understanding the efficacy and safety outcomes discussed in subsequent sections.

## 4. Clinical Efficacy of Aflibercept and Brolucizumab in DME in Randomized Controlled Trials

Aflibercept has been extensively evaluated for the treatment of diabetic retinopathy and diabetic macular edema, with robust evidence derived from large randomized controlled trials and long-term follow-up studies. Its clinical efficacy has been demonstrated across a broad spectrum of disease severity, including patients with significant vision loss and advanced diabetic retinopathy.

The pivotal VIVID and VISTA trials established the efficacy of aflibercept in patients with diabetic macular edema, demonstrating significant improvements in best-corrected visual acuity compared with laser photocoagulation [22]. These trials also reported marked reductions in central retinal thickness, highlighting the strong anatomical response associated with aflibercept therapy. Importantly, visual and anatomical benefits were sustained over extended follow-up with continued treatment [22,23,24], supporting the long-term use of aflibercept in chronic diabetic retinal disease (Table 1).

Comparative data from the DRCR.net Protocol T study further reinforced the role of aflibercept in DME management [25,26]. In this trial, aflibercept demonstrated greater visual acuity gains in eyes with worse baseline visual acuity compared with other anti-VEGF agents, particularly during the first year of treatment [26]. These findings underscored the importance of baseline disease characteristics in treatment selection and positioned aflibercept as a preferred option in patients with more severe visual impairment.

Beyond improvements in visual acuity and retinal thickness, aflibercept has been associated with favorable effects on diabetic retinopathy severity. Post hoc analyses of randomized trials VIVID and VISTA have reported significant improvements in Diabetic Retinopathy Severity Scale scores, including ≥2-step DRSS improvement in a proportion of treated eyes, suggesting potential disease-modifying effects [23,39].

Long-term extension studies and real-world observational data, including large prospective cohorts such as APOLLON [29,30] and multinational datasets like AURIGA [35], have supported the durability and consistency of aflibercept outcomes over time, confirming the efficacy observed in randomized clinical trials, such as VIVID, VISTA [22,23,24], and Protocol T [25,26]. Visual gains and anatomical improvements were generally maintained with ongoing therapy, although the need for regular intravitreal injections highlights the persistent treatment burden associated with anti-VEGF therapy in routine clinical practice [32,33,34,35,36]. Across real-world observational studies—including post-marketing surveillance in Japan [34], retrospective multicenter European and Turkish cohorts [33], and single-center analyses [32,36]—patients generally achieve visual and anatomical benefits with fewer injections and less intensive monitoring than in RCTs, reflecting adherence variability, healthcare access, and individualized treatment strategies. Moreover, emerging evidence from high-dose aflibercept (8 mg) studies, including PHOTON Phase 2/3 and extension trials [37,38], indicates that higher-dose regimens allow extended dosing intervals while maintaining BCVA and CST improvements. The 8 mg formulation demonstrates non-inferior efficacy to 2 mg, with fewer injections, improved durability in refractory eyes, and maintained safety over long-term follow-up, although persistent fluid may occur in some cases. The overall safety profile of aflibercept 8 mg was comparable to that of the 2 mg formulation, with no new ocular or systemic adverse events identified. Rates of common ocular treatment-emergent adverse events, such as transient intraocular pressure elevation, conjunctival hemorrhage, or mild intraocular inflammation, were similar between doses, and serious complications—including retinal vasculitis, occlusive retinitis, or endophthalmitis—were not observed at higher frequencies. However, more evidence is needed to document the safety profile of higher doses and identify patients most likely to benefit from high-dose therapy and optimizing individualized dosing intervals.

Brolucizumab is a newer anti–vascular endothelial growth factor agent that has been evaluated for the treatment of diabetic macular edema and diabetic retinopathy with a particular focus on treatment durability and sustained disease control. Its clinical efficacy has been primarily established through large randomized controlled trials, most notably the KESTREL and KITE studies [40,41]; Table 2.

The KESTREL and KITE trials demonstrated that brolucizumab achieved non-inferior improvements in best-corrected visual acuity (BCVA) compared with aflibercept in patients with diabetic macular edema (DME) [40,41]. Visual outcomes were maintained throughout the study periods, confirming the ability of brolucizumab to effectively preserve vision in this patient population.

In terms of anatomical outcomes, brolucizumab was associated with robust reductions in central retinal thickness (CRT), with several analyses reporting numerically greater retinal drying compared with aflibercept [40,41]. These anatomical findings have been attributed to the high molar dose and strong VEGF-A binding affinity of brolucizumab, which may facilitate more complete and sustained VEGF suppression within the retina. A key distinguishing feature of brolucizumab therapy is its potential for extended dosing intervals. In both KESTREL and KITE, a substantial proportion of patients were maintained on 12-week dosing intervals following the loading phase, highlighting the possibility of reduced injection frequency in selected individuals [11,40]. This durability represents a potential advantage in managing a chronic condition such as DME, where treatment burden and suboptimal adherence are major considerations.

Beyond macular edema control, brolucizumab has also demonstrated efficacy in improving diabetic retinopathy severity. Post hoc analyses reported meaningful improvements in Diabetic Retinopathy Severity Scale (DRSS) scores, including regression of retinopathy in a subset of treated eyes, suggesting that brolucizumab, similar to other anti-VEGF agents, may exert disease-modifying effects in diabetic retinopathy. Additional subgroup and durability analyses have provided further insight into patient selection for brolucizumab therapy, indicating that patients achieving early disease control are more likely to maintain stable visual and anatomical outcomes on extended dosing regimens [40,41,42,43,44,45,46].

Post hoc analyses of KESTREL and KITE showed that patients achieving disease control within the first three months were more likely to maintain stable BCVA and CRT at 52 weeks [40] and up to 100 weeks [41]. The KINGFISHER trial similarly found that early responders experienced sustained anatomical and functional benefits [42]. Real-world studies further support these findings: Bragança et al. [43] and the BRADIR study by Chakraborty et al. [44] reported that patients with early disease control were more likely to maintain extended dosing intervals and avoid treatment intensification. Comparative real-world analyses with aflibercept suggest that early response may predict longer-term stability, with one-year outcomes showing better visual and anatomical maintenance in early responders versus those with delayed response [45,46]. Additionally, a recent meta-analysis of randomized controlled trials comparing brolucizumab and aflibercept confirmed slightly greater reductions in central subfield thickness with brolucizumab over 52–100 weeks, supporting the notion that early anatomical response may translate into sustained long-term benefits [53].

Collectively, these data indicate that early anatomic and functional control of DME may be a useful predictor of long-term outcomes and can inform individualized treatment planning with anti-VEGF agents. Real-world observational studies further support these findings, showing BCVA improvement or stabilization and significant CRT/CST reduction across treatment-naïve and switch populations, including early extension of treatment intervals and reduced injection burden [43,44,45,46,47,49,50,51]. Notably, PRN dosing of brolucizumab in early-onset, treatment-naïve DME also demonstrated visual and anatomical benefits while allowing flexible treatment intervals [52].

However, interpretation of these efficacy outcomes must be balanced with consideration of the safety profile of brolucizumab, which is discussed in detail in the following section.

## 5. Comparative Safety Profiles of Intravitreal Aflibercept vs. Brolucizumab

Safety is a critical consideration in the long-term management of diabetic retinopathy and diabetic macular edema, given the chronic nature of the disease and the need for repeated intravitreal injections. Both aflibercept and brolucizumab demonstrated acceptable overall safety profiles in randomized clinical trials; however, clinically meaningful differences have emerged, particularly with regard to intraocular inflammatory events. In pivotal clinical trials evaluating aflibercept for DME, ocular adverse events were generally infrequent and consistent with those expected following intravitreal anti-VEGF therapy. Reported events included conjunctival hemorrhage, transient elevations in intraocular pressure, and mild intraocular inflammation, with low rates of serious ocular complications [22,23,24,25,26,27,28]. Long-term follow-up studies and real-world observational data have further supported the favorable and well-characterized safety profile of aflibercept, reinforcing its reliability in routine clinical practice [23,24,26,30,31,32,33,34,35,36,37,38].

In contrast, safety concerns related to brolucizumab became more apparent following its broader clinical use. Although the KESTREL and KITE trials reported low overall rates of ocular adverse events and comparable systemic safety outcomes to aflibercept [40,41], post-marketing surveillance and real-world data identified cases of intraocular inflammation, retinal vasculitis (RV), and retinal vascular occlusion (RO) associated with brolucizumab treatment [54,55,56,57]. These reports indicate that immune-mediated intraocular inflammation may occur with or without concomitant RV and can present at variable time points following injection [41,47]. RO, although rare, has been identified as a particularly serious adverse event, underscoring the importance of prompt recognition and management [55,56,57]. While these events were relatively uncommon, their potential severity and association with visual loss prompted heightened clinical awareness.

Witkin et al. [58] critically analyzed a case series of 25 patients, mostly women (88%), experiencing retinal vasculitis after intravitreal brolucizumab. The analysis was based on the cases reported to the American Society of Retina Specialists (ASRS), after approval of brolucizumab. All patients were previously treated with other anti-VEGFs for wet macular degenerescence, with no history of ocular inflammation. The symptoms varied from mild floaters to severe decreased vision, and the vasculitis was developed in a rather delayed fashion. The mainstay therapy is based on corticosteroids, applied topical, subtenonian or intravitreal, according to severity and doctor’s decision. The authors draw attention of careful follow-up for any signs of ocular inflammation after brolucizumab injection, as well as avoid its use in monocular patients, or when planning bilateral injections on the same moment. Similar findings were reported in a multicentric case-series of Chamal et al. [59], involving 12 patients from 10 centers in United States. These reports ended in a general consensus recommendation of not performing intravitreal brolucizumab earlier than the 8-week interval to decrease the risk of IOI in neovascular macular degenescence [60].

For unknown reasons, however, the cases of IOI and vasculitis appear to be less frequent in patients with DME [14]. Registry-based analyses and retrospective observational studies have reported heterogeneous incidence rates and clinical presentations, reflecting differences in patient selection, monitoring strategies, and treatment practices [42,61,62,63,64,65]. Some reports suggest that early diagnosis and timely initiation of corticosteroid therapy may mitigate visual consequences in affected patients; however, visual outcomes following severe inflammatory events remain variable, underscoring the need for cautious patient selection and vigilant follow-up [41,42,62,63,64,65]; Table 3.

Across treatment-naïve and switch populations, systemic adverse events were rare or absent, and no new safety signals were reported [43,44,45,46,47,48,49,50,51,52]. Ocular adverse events, including intraocular inflammation and retinal vasculitis, were uncommon and generally mild or manageable without permanent vision loss. Rare events of retinal vascular occlusion were reported, but overall incidence remained very low. Hirano et al. [47] reported an incidence of 7.1% of IOI, and 1.8% (one case with bilateral injection) for RV. Hansraj et al. in a study involving 13 patients, reported 1 case of IOI (7.6%), but no cases or retinal vasculitis. However, all cases responded well to prompt steroid therapy. Other real-world studies on smaller number of patients do not report significant adverse events related to intravitreal brolucizumab [43,44,45,46,49,51,52]. These findings indicate that brolucizumab can be safely administered in routine clinical practice, including in extended-interval or PRN dosing regimens, while maintaining vigilance for inflammatory events.

Igwe et al. [65] found that IOI-immune related to intravitreal Brolucizumab was encountered more frequently in 6 mg q4 week arm (9.3%) compared with the brolucizumab 6 mg q8/q12 week arms (4.4%) in the Phase 3 clinical studies [66]. Multiple studies showed that IOI events may develop any time after treatment, but there are more frequent in the first 6 months after the initiation, with a peak incidence is around day 25–26 after injection [40,65,66,67,68,69,70]. The most relevant risk factors were female sex, and a personal history of intraocular inflammation and/or retinal vascular occlusion in the year prior to treatment. A recent study pointed out that patients who experience IOI develop anti-drug specific antibodies. However, only 2.1% of these patients will experience severe adverse events, such as RV and RO, indicating a more complex mechanism is involved [67]. Early recognition of the signs of ocular inflammation and prompt steroid therapy is extremely important to prevent vision loss [60,67,68,69]. As a result, brolucizumab use in DME has often been reserved for carefully selected patients, such as those with high treatment burden or suboptimal response to other anti-VEGF agents, with emphasis on informed consent and close post-injection monitoring. Informed consent after a reasonable disclosure and patient education about possible adverse effect should be not overlooked [69,70,71].

In summary, while both aflibercept and brolucizumab are effective anti-VEGF therapies for diabetic retinal disease, their safety profiles differ in clinically important ways. Aflibercept is supported by extensive long-term safety data and a predictable adverse event profile, whereas brolucizumab offers potential advantages in durability at the expense of an increased risk of intraocular inflammatory events. These safety considerations are central to individualized treatment selection and inform the clinical decision-making discussed in the subsequent sections.

## 6. Treatment Burden and Real-World Evidence

The long-term management of diabetic retinopathy and diabetic macular edema requires sustained therapy and frequent monitoring, making treatment burden a critical determinant of real-world outcomes [71,72]. While randomized controlled trials provide essential evidence regarding efficacy and safety, real-world studies offer complementary insights into adherence, durability, and treatment performance in broader and more heterogeneous patient populations.

Aflibercept has been widely evaluated in real-world settings, with multiple observational studies confirming the visual and anatomical benefits observed in clinical trials [9,22,23,24,25,26,27,28,29,30,31,32,33,34,35,36,37,38]. These studies have reported meaningful improvements in best-corrected visual acuity and reductions in central retinal thickness when aflibercept is administered in everyday practice. However, real-world data have also highlighted the challenges of maintaining frequent injection schedules over time, with under-treatment commonly reported due to patient-related, logistical, and healthcare system factors [11,46,70,71,72,73]. Despite its established efficacy, aflibercept often requires regular intravitreal injections at relatively short intervals, which may negatively affect adherence and long-term outcomes in some patients [11,35,36,37,38,74]. These findings underscore the gap between clinical trial protocols and real-world treatment patterns and emphasize the importance of individualized dosing strategies.

Brolucizumab has attracted interest in real-world practice largely due to its potential for extended dosing intervals. Observational studies and post-marketing reports suggest that a subset of patients can achieve satisfactory anatomical control with reduced injection frequency, particularly those switched from other anti-VEGF agents because of persistent or recurrent macular edema [44,46,47,48]. Switching studies have described improvements in retinal thickness following transition to brolucizumab in patients with refractory disease. However, these potential benefits must be interpreted cautiously, given the heterogeneity of study designs, short follow-up durations, and the need for careful safety monitoring [46,47,48]. Real-world safety considerations have strongly influenced the clinical use of brolucizumab. Heightened awareness of intraocular inflammation and retinal vasculitis has led to more selective prescribing and closer post-injection surveillance in routine practice. In contrast, the extensive real-world experience with aflibercept has reinforced its reputation as a reliable and predictable treatment option across diverse patient populations [11,74,75,76].

In addition to efficacy and safety considerations, economic factors and patient access should be acknowledged when comparing aflibercept and brolucizumab. Both agents are high-cost biologic therapies requiring repeated intravitreal administration, and cumulative treatment expenses may impose a substantial financial burden on healthcare systems and patients. Recent literature highlights the broader implications of rising drug costs on formulary positioning, reimbursement decisions, and health system sustainability [77]. Moreover, cost-related nonadherence and variability in insurance coverage have been shown to significantly affect treatment continuity and real-world utilization patterns [78]. Evidence from health services research further indicates that out-of-pocket expenses and access disparities can influence equitable implementation of evidence-based therapies [78]. Therefore, consideration of pricing structures, reimbursement policies, and patient affordability is essential for contextualizing the real-world applicability of these therapeutic options.

Importantly, real-world clinical experience, including previously published institutional data, has highlighted the need for flexible and patient-centered treatment approaches that account for disease severity, prior treatment response, and individual risk profiles [43,44,45,46,47,48]. Such evidence illustrates the complexity of translating trial-based regimens into daily practice and supports the role of individualized treatment strategies in optimizing long-term outcomes.

## 7. Clinical Considerations, Limitations and Future Perspectives

### 7.1. Clinical Considerations and Patients’ Selection

The selection of anti–vascular endothelial growth factor therapy for patients with diabetic retinopathy and diabetic macular edema requires an individualized approach that balances efficacy, safety, treatment burden, and patient-specific factors. Although both aflibercept and brolucizumab have demonstrated strong anti-VEGF activity, differences in pharmacological characteristics, durability, and safety profiles necessitate careful consideration in clinical practice [7,8,9,10,11,12,13,14,15,16].

Aflibercept remains a widely adopted first-line therapy for many patients with DME, supported by extensive randomized trial data and long-term real-world experience demonstrating predictable efficacy and a well-established safety profile [22,23,24,25,26,27,28,29,30,31,32,33,34,35,36,37,38]. It is often favored in patients with advanced disease, significant visual impairment, or comorbidities where treatment safety and reliability are prioritized.

Brolucizumab may be considered in selected patients, particularly those with persistent or recurrent macular edema despite prior anti-VEGF therapy or those experiencing a high treatment burden. The potential for extended dosing intervals represents a meaningful advantage for certain individuals, especially when frequent clinic visits are challenging. However, though the results may suggest that brolucizumab is better in durability, more evidence is needed to sustain this idea. The trials compared brolucizumab 12w vs. aflibercept 8w. The design does not indicate if alibercept 12w is inferior to brolucizumab 12w or not. Moreover, given the reported risk of intraocular inflammation and retinal vasculitis, careful patient selection, informed consent, and close post-injection monitoring are essential when initiating brolucizumab therapy [44,45,46,47,48].

Baseline disease characteristics, including visual acuity, retinal thickness, and diabetic retinopathy severity, play an important role in treatment selection and response. Patients with more severe disease or poorer baseline vision may benefit from agents with robust and predictable efficacy, whereas patients with stable disease and good treatment response may prioritize durability and reduced injection frequency [11,13,14,15,16]. Patient preferences, lifestyle factors, and access to care should be integrated into shared decision-making to optimize adherence and outcomes.

Patient selection and risk stratification play an important role in optimizing anti-VEGF therapy in diabetic retinal disease. Consideration of factors such as prior response to anti-VEGF treatment, extent of retinal ischemia, monocular status, and individual risk tolerance may help guide agent selection. Patients with limited ability to adhere to frequent follow-up or a high perceived treatment burden may benefit from therapies offering greater durability, whereas those with advanced disease or heightened concern regarding inflammatory complications may warrant agents with more established long-term safety profiles. Integrating patient preferences, systemic comorbidities, and capacity for ongoing monitoring into shared decision-making is essential for achieving sustainable long-term disease control.

Regular assessment of visual function, anatomical response, and signs of inflammation is critical, particularly when using agents associated with inflammatory risk. Prompt recognition and management of adverse events can mitigate potential vision-threatening complications and improve overall outcomes. Ultimately, the choice between aflibercept and brolucizumab should be guided by a comprehensive evaluation of clinical evidence and individualized patient needs. Rather than a one-size-fits-all approach, personalized treatment strategies that adapt over time in response to disease activity and patient circumstances are likely to yield the best long-term results in diabetic retinal disease.

### 7.2. Limitations of Current Evidence

Despite the substantial body of evidence supporting the use of anti-VEGF therapy in diabetic retinopathy and diabetic macular edema, several limitations of the current literature should be acknowledged. Direct head-to-head comparisons between aflibercept and brolucizumab in diabetic retinal disease remain limited. Although the KESTREL and KITE trials provide valuable comparative data, differences in trial design, patient populations, retreatment criteria, and outcome measures complicate direct cross-trial comparisons and limit definitive conclusions regarding relative efficacy and durability [40,41,42]. Randomized controlled trials may not fully reflect real-world clinical settings. Strict inclusion and exclusion criteria often result in study populations that differ from those encountered in everyday practice, where patients frequently present with multiple comorbidities, variable adherence, and heterogeneous disease characteristics. While real-world studies help address this gap, they are inherently subject to selection bias and variability in treatment regimens [9,45,46,47,48].

Follow-up durations in several clinical trials and observational studies are relatively limited, particularly with respect to long-term safety outcomes. Although aflibercept benefits from extensive long-term safety data, the longer-term safety profile of brolucizumab—especially regarding inflammatory events—continues to be defined through ongoing post-marketing surveillance and real-world reporting. Additionally, heterogeneity in outcome measures, including differences in visual acuity endpoints, anatomical assessments, and definitions of disease improvement, poses challenges for evidence synthesis. Variations in monitoring protocols and retreatment strategies further contribute to inconsistencies across studies and may influence reported outcomes [8,9,10,11,12,15,16,17].

Finally, publication bias and the rapid evolution of therapeutic strategies may affect the available evidence base. As new agents and treatment paradigms emerge, previously published data may not fully reflect contemporary clinical practice, underscoring the need for ongoing research and updated analyses [79,80,81].

Taken together, these limitations highlight the importance of cautious interpretation of the literature and reinforce the need for individualized treatment strategies guided by both evidence and clinical judgment.

### 7.3. Future Directions

The therapeutic landscape for diabetic retinopathy and diabetic macular edema continues to evolve, with ongoing efforts aimed at improving treatment durability, safety, and real-world feasibility. Future research will play a central role in refining the optimal use of existing anti-VEGF agents and in defining the place of emerging therapies within increasingly individualized treatment strategies. Longer-term comparative studies are needed to better characterize the relative durability and safety profiles of aflibercept and brolucizumab, particularly in real-world clinical settings. Extended follow-up data will be especially important for further elucidating the incidence, risk factors, and long-term visual outcomes associated with inflammatory events reported with brolucizumab, as well as for confirming the long-term safety of extended dosing strategies [79,80,81].

Advances in retinal imaging and biomarker development may facilitate more personalized treatment approaches. The identification of anatomical, functional, or molecular predictors of treatment response could help guide agent selection, optimize dosing intervals, and reduce unnecessary injections. Such precision-medicine strategies may be particularly valuable in managing patients with heterogeneous disease phenotypes and variable responses to therapy [80,81,82,83].

In parallel, the development of longer-acting anti-VEGF agents and novel drug delivery systems holds promise for reducing treatment burden in chronic retinal disease. Sustained-release formulations, alternative molecular targets, and combination therapies addressing both angiogenic and inflammatory pathways are under active investigation and may further improve long-term disease control [81,82,83].

Finally, real-world evidence and pragmatic clinical studies will remain essential for understanding how both established and emerging therapies perform outside controlled trial environments. Incorporating patient-reported outcomes, quality-of-life measures, and health-economic analyses into future research will further inform clinical decision-making and healthcare policy in the management of diabetic retinal disease [82,83].

## 8. Conclusions

Anti-VEGF therapy has transformed the management of DME, offering substantial visual and anatomical benefits for a broad range of patients. Among available agents, aflibercept and brolucizumab represent effective treatment options with distinct pharmacological properties, clinical profiles, and safety considerations. Aflibercept is supported by a robust body of randomized trial and real-world evidence demonstrating predictable efficacy and a well-established safety profile, making it a cornerstone therapy in clinical practice. Brolucizumab has demonstrated comparable efficacy with the potential advantage of extended dosing intervals in selected patients, though its use is tempered by concerns regarding intraocular inflammation and retinal vasculitis.

Careful interpretation of the available evidence highlights the importance of individualized treatment selection that accounts for disease severity, treatment burden, patient preferences, and safety considerations. Rather than favoring a single agent universally, flexible and patient-centered approaches are essential for optimizing long-term outcomes in diabetic retinal disease.

## Figures and Tables

**Table 1:** Clinical Trials involving Aflibercept in DME.

Study (Author, Year)	Study Type	Population (Number of Patients; Treatment-Naïve/Switch)	Anti-VEGF Agent (Dosing Regimen)	Follow-Up Time	BCVA Outcome (Change)	Anatomical Outcome	Durability/Injection Burden
VIVID/VISTA [22] (Korobelnik et al., 2014)	Phase III, randomized, double-masked RCTs	CI-DME; *n* = 872; treatment-naïve	Aflibercept 2 mg q4w or q8w (after loading) vs. laser	52 weeks	Significant BCVA gains vs. laser	Significant CST reduction vs. laser	q8w dosing maintained efficacy with fewer injections than q4w
VIVID/VISTA [23] (Brown et al., 2015)	Phase III RCT report (extended follow-up)	CI-DME; VIVID/VISTA cohorts	Aflibercept 2 mg q4w or q8w vs. laser	100 weeks	Visual benefits maintained; continued superiority vs. laser	Anatomical benefits maintained	Sustained efficacy through 2 years
VIVID/VISTA [24] (Heier et al., 2016)	Phase III RCT report (3-year results)	CI-DME; *n* = 872	Aflibercept 2 mg q4w or q8w vs. laser	148 weeks	Sustained visual improvements vs. laser	Sustained CST reduction	Durable efficacy and safety over 3 years
Protocol T [25] (Wells et al., 2015)	Multicenter randomized comparative-effectiveness RCT	CI-DME; *n* ≈ 660; treatment-naïve	Aflibercept vs. ranibizumab vs. bevacizumab (protocol-driven retreatment)	52 weeks	All improved; aflibercept showed greater gains in worse baseline VA subgroup	CST reduced across groups	Comparative evidence supporting aflibercept as preferred option in worse baseline VA
Protocol T [26] (Wells et al., 2016)	RCT (2-year outcomes)	CI-DME; Protocol T cohort	Aflibercept vs. ranibizumab vs. bevacizumab	104 weeks	Gains maintained; subgroup differences attenuated	Sustained anatomical improvement	Fewer injections in year 2 while maintaining outcomes (protocol-driven)
ENDURANCE extension after VISTA [27] (Wykoff et al., 2018	Extension study (PRN)	Prior VISTA participants	PRN aflibercept (with/without laser)	12 months extension	Vision gains generally maintained	Anatomic control maintained	Reduced injection frequency; subset required no injections during extension
Protocol V [28] (Baker et al., 2019)	Multicenter RCT (initial strategy)	CI-DME with good baseline VA; *n* = 702; treatment-naïve	Initial aflibercept vs. laser vs. observation (rescue aflibercept if VA worsened)	104 weeks	No significant difference in vision loss at 2 years among strategies	CST changes reported (strategy-dependent)	Many eyes managed without immediate injections; rescue therapy required in a subset
APOLLON [29] (Korobelnik et al., 2020)	Prospective real-world observational study	DME; *n* = 402; treatment-naïve + previously treated	Aflibercept monotherapy (routine practice)	12 months	Meaningful BCVA improvement in real-world practice	CST reduction in real-world practice	Real-world injection frequency lower than RCT protocols
APOLLON [30] (Korobelnik et al., 2022)	Prospective real-world observational study (extension)	DME; *n* = 402 (extension cohort analyzed)	Aflibercept monotherapy	24 months	Functional outcomes maintained/improved over longer follow-up	Anatomical improvements maintained	Sustained real-world outcomes with individualized dosing
Kim et al., 2020 [31]	Prospective, multicenter interventional (single-arm)	DME; *n* = 48	Aflibercept T&E with interval adjustment	104 weeks	BCVA improvement maintained on T&E	CST reduction maintained	Extended injection intervals achievable with maintained outcomes
Lukic et al., 2020 [32]	Retrospective real-world cohort	DME; *n* ≈ 99 eyes; treatment-naïve	Aflibercept; routine dosing	12 mo	BCVA improved (~+10 letters)	CST reduced (~431→306 µm)	Mean 6.9 injections in year 1 (lower than RCTs)
Kucukevcilioglu et al., 2023 [33]	Retrospective comparative real-world	DME; *n* ≈ 212 (aflibercept arm)	Aflibercept vs. ranibizumab	24 months	Comparable BCVA gains	Similar CST reduction	Fewer injections with aflibercept
Sugimoto et al., 2022 [34]	Prospective observational (post-marketing)	DME; *n* = 646; mixed population	Aflibercept 2 mg (real-world use)	24 months	Significant BCVA improvement	CST reduction	Fewer injections vs. RCTs
AURIGA [35] (Donati, S.; 2024)	Prospective multinational observational	DME (mixed retinal cohort)	Aflibercept (individualized)	24 months	Sustained functional improvement	Sustained anatomical control	Flexible dosing; reduced burden
Barbosa et al., 2025 [36]	Retrospective real-world study	DME; *n* = 67 (100 eyes)	Aflibercept 8 mg	~6 months	VA stabilization	Significant CST reduction	Some extended intervals; variable durability
PHOTON [37] (Brown et al., 2024)	Randomized, double-masked, Phase 2/3 RCT	CI-DME; *n* ≈ 658	Aflibercept 8 mg q12 or q16 vs. 2 mg q8	48–96 weeks	Non-inferior BCVA gains vs. 2 mg	Comparable CST reduction	Extended intervals, fewer injections
PHOTON Extension Study [38] (Do et al., 2025)	Open-label extension	CI-DME	Aflibercept 8 mg	~156 weeks	Maintained BCVA improvement	Sustained CST reduction	Durable effect with extended dosing

BCVA, best-corrected visual acuity; CST, central subfield thickness; CI-DME, center-involving diabetic macular edema; RCT, randomized controlled trial; PRN, pro re nata; T&E, treat-and-extend; VA, visual acuity.

**Table 2:** Clinical Trials involving Brolucizumab vs. Aflibercept and Brolucizumab only in DME.

Study (Author, Year)	Study Type	Population (Number of Patients; Treatment-Naïve/Switch)	Anti-VEGF Agent (Dosing Regimen)	Follow-Up Time	BCVA Outcome (Change)	Anatomical Outcome	Durability/Injection Burden
KESTREL & KITE (Brown et al., 2022) [40]	Phase III, randomized, double-masked, non-inferiority RCTs	DME; *n* = 1058 (KESTREL *n* ≈ 566; KITE *n* ≈ 492); treatment-naïve	Brolucizumab 6 mg q12w/q8w vs. aflibercept 2 mg q8w (after loading)	52 weeks	Mean BCVA gains non-inferior to aflibercept	Greater reduction in CST with brolucizumab in both trials	A substantial proportion of patients maintained on q12w dosing, indicating reduced injection burden
KESTREL & KITE (Wykoff et al., 2024) [41]	Phase III, randomized, double-masked, non-inferiority RCTs	DME; *n* = 1058 (KESTREL *n* ≈ 566; KITE *n* ≈ 492); treatment-naïve	Brolucizumab 6 mg q12w/q8w vs. aflibercept 2 mg q8w	100 weeks	BCVA gains maintained over 100 weeks and non-inferior to aflibercept	Sustained and numerically greater reductions in CST with brolucizumab in several analyses	A high proportion of patients remained on extended q12w dosing, supporting long-term durability
KINGFISHER (Singh et al., 2023) [42]	Randomized, double-masked clinical trial	DME; *n* = 178; treatment-naïve	Brolucizumab 6 mg q8w/q12w vs. aflibercept 2 mg q8w	52 weeks	Comparable mean BCVA improvements between brolucizumab and aflibercept	Greater reductions in CST observed with brolucizumab at multiple time points	Potential for extended dosing intervals with brolucizumab, suggesting reduced injection burden
Bragança et al. (2024) [43]	Retrospective, real-world observational study	DME; *n* = 32; treatment-naïve and previously treated	Brolucizumab 6 mg (treat-and-extend in clinical practice)	12 months	Improvement in mean BCVA from baseline during follow-up	Significant reduction in CRT observed	Extended treatment intervals achieved in many patients, suggesting reduced injection burden
Chakraborty et al. (2022) [44]	Prospective real-world observational study	Chronic center-involving DME; *n* = 12; previously treated (switch)	Brolucizumab 6 mg with early extended-interval regimen	6 months	Mean BCVA improved or stabilized from baseline during follow-up	Reduction in CMT observed after switching to brolucizumab	Early extension of treatment intervals achieved in most patients, indicating reduced injection burden
Elhamaky et al. (2024) [45]	Retrospective real-life comparative case series	Treatment-naïve center-involving DME; *n* = 44; naïve	Brolucizumab 6 mg vs. aflibercept 2 mg (real-world dosing)	12 months	Comparable improvement in mean BCVA between treatment groups at 1 year	Greater reduction in CMT observed in the brolucizumab group	Longer treatment intervals achieved with brolucizumab in routine practice
Rübsam et al. (2024) [46]	Retrospective real-world comparative cohort study	DME; *n* = 191; treatment-naïve and therapy-refractory (switch)	Brolucizumab 6 mg vs. aflibercept 2 mg (treat-and-extend in routine practice)	12 months	Improvement in mean BCVA from baseline in both groups, with no significant inter-group difference	Greater and more sustained reduction in CRT with brolucizumab	Longer treatment intervals and fewer injections achieved with brolucizumab in a subset of patients
Hirano et al. (2025) [47]	Retrospective, single-center observational study	DME; *n* = 47; previously treated (switch from other anti-VEGF agents)	Brolucizumab 6 mg (after prior anti-VEGF therapy)	6 months	Improvement or stabilization of BCVA observed after switching to brolucizumab	Significant reduction in CRT following treatment switch	Extension of treatment intervals achieved in a proportion of patients, suggesting reduced injection burden
KINGFISHER post hoc (Ip et al., 2025) [48]	Post hoc analysis of randomized clinical trial	DME; *n* ≈ 178; treatment-naïve and previously treated (switch)	Brolucizumab 6 mg q8w/q12w (subgroup analysis by prior anti-VEGF exposure)	52 weeks	Comparable BCVA improvements observed in both treatment-naïve and switch subgroups	Consistent reduction in CRT across subgroups	Extended dosing intervals maintained in both naïve and switch patients, supporting reduced injection burden
Chronopoulos et al., 2025 [49]	Prospective real-world cohort	DME; *n* = 33; naïve + refractory	Brolucizumab 6 mg	6–12 months	Significant BCVA improvement	Significant CST reduction; improved perfusion	Real-world intervals with functional/anatomical benefit
Hansraj et al., 2025 [50]	Prospective interventional real-world	Persistent DME; *n* = 19	Brolucizumab 6 mg	~13 weeks	Median VA improved	CRT reduced ~54%	Mean reinjection interval ~11 weeks
Pastore et al., 2024 [51]	Retrospective real-world	Switched DME eyes; *n* = 28	Brolucizumab 6 mg	6–12 months	BCVA improved	CST/structural OCT changes	Many patients achieved extended q12-week dosing
Bilgic et al., 2025 [52]	Prospective real-world study	Early onset, treatment-naïve DME; n not specified	Brolucizumab PRN	6–12 months	BCVA improved	Significant CRT reduction	PRN regimen allowed flexible dosing, reducing injection burden

**Table 3:** Safety and Inflammatory Events Associated with intravitreal Brolucizumab vs. Aflibercept in DME in RCT trials.

Safety Domain	Study/Source	Brolucizumab	Aflibercept
Systemic adverse events	KESTREL, KITE, KINGFISHER [40,41,42]	Comparable; low incidence	Comparable; low incidence
Overall ocular adverse events	KESTREL/KITE pooled [40,41]	3.7–4.2%	1.1–2.1%
	Hirano [47]	8.70%	
Intraocular inflammation (IOI)	KINGFISHER [42]	4.0%	2.9%
	KESTREL [41]	4.2%	1.1%
	KITE [41]	2.2%	1.7%
	Hirano [47]	7.10%	
	Hansraj [50]	7.60%	
Retinal vasculitis	KINGFISHER [42]	0.9%	0.6%
	KESTREL [41]	0.5%	0%
	KITE [41]	0%	0%
	Hirano [47]	1.70%	
	Hansraj [50]	0%	
Retinal vascular occlusion	KINGFISHER [42]	0.3%	0.6%
	KESTREL [41]	1.6%	0.5%
	KITE [41]	0.6%	0.6%

## Data Availability

Data sharing is not applicable to this article as no new data were generated or analyzed.

## Abbreviations

BCVA	Best-corrected visual acuity
CRT	Central retinal thickness
DME	Diabetic macular edema
DR	Diabetic retinopathy
DRSS	Diabetic Retinopathy Severity Scale
IOI	Intraocular inflammation
RCT	Randomized controlled trial
RO	Retinal vascular occlusion
RV	Retinal vasculitis
VEGF	Vascular endothelial growth factor
